# Supplementary material for: Genetic Transformation of Quercus ilex Somatic Embryos with a Gnk2-like Protein That Reveals a Putative Anti-Oomycete Action
Source: Plants (Basel). 2022 Jan 24;11(3):304. doi: 10.3390/plants11030304 (PMC8838351; doi:10.3390/plants11030304)
Supplement: Supplementary file 1 [file plants-11-00304-s001.zip › plants-1548723-supplementary.pdf]

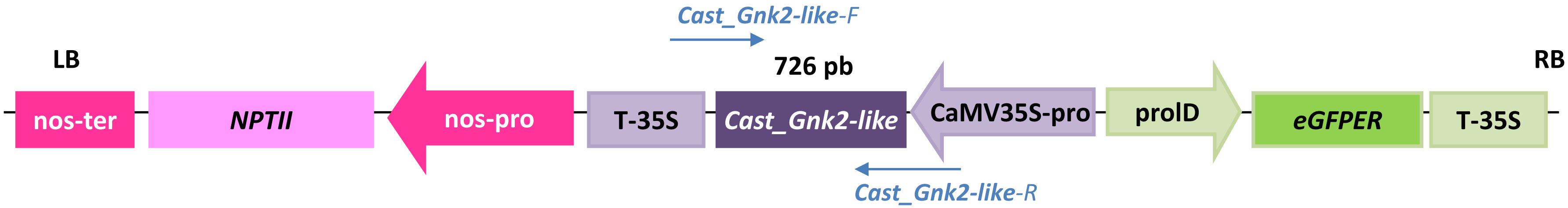


**Online resource 1.** A schematic representation of T-DNA region of the plasmid pK7WG2D-Gnk2. *nos-ter*, *nos-pro* terminator and promoter of nopaline synthase gene, respectively; *NPTII* neomycin phosphotransferase marker gene; CaMV*35S-pro, T-35S* promoter and terminator of Cauliflower mosaic virus gene, respectively; *eGFPER* green fluorescence protein gene*; Cast_Gnk2-like* gene encoding a Ginkbilobin-2 protein from *C. crenata*; *prolD* rol root loci D promoter; *RB* right border; *RB* left border.

e

**Online resource 2.** Primers and amplification programs utilized in the present report.

| GENE or PROMOTER | PRIMER NAME | PRIMER SEQUENCE (5′-3′) | PCR CONDITIONS | FRAGMENT AMPLIFIED (bp) | PURPOSE | qPCR Efficiency (%) and $R^2$ |
|---|---|---|---|---|---|---|
| NPTII | NPTII-F<br>NPTII-R | GTCATCTCACCTTGCTCCTGCC<br>AAGAAGGCGATAGAAGCGA | 35 cycles:<br>94ºC x 30s<br>60ºC x 30s<br>72ºC x 42s | 472 | PCR analysis | - |
| GFP | EGFP-F<br>EGFP-R | CACCGGGGTGGTGCCCAT<br>CTAGTGGATCCCCCGGGC | 40 cycles:<br>94ºC x 15s<br>56ºC x 30s<br>72ºC x 1min | 740 | PCR analysis | - |
| Cast_Gnk2-like-F[1] | T35S-R<br>GIN-D | AGGTCACTGGATTTTGGT<br>CTGCCACTAGCCGTTATGGT | 35 cycles:<br>98ºC x 10s<br>56ºC x 30s<br>72ºC x 1min | 890 | PCR analysis | - |
| Cast_Gnk2-like-R[2] | p35S-D<br>GIN-R | GATCTAACAGAACTCGCC<br>CTGGTGCATTGAGCCAAACC | 35 cycles:<br>98ºC x 10s<br>56ºC x 30s<br>72ºC x 1min | 1227 | PCR analysis | - |
| CaMV35S | P35S-F<br>P35S-R | GGACGATTCAAGGCTTGCT<br>AGTCTTCACGGCGAGTTCT | Tm 58ºC | 137 | qPCR copy number | See Results |
| Cast_Gnk2-like | Cc_Gnk2-F<br>Cc_Gnk2-R | GGGGACCTAAAGCTTGACTCA<br>CATCGCAACAGTTGGGAAGTT | Tm 62ºC | 129 | Transgene expression for qPCR | 94; 0.991 |
| EF1α | EF1α-F<br>EF1α-R | GTGCCGTCCTCATTATTGAC<br>CACGGGTCTGACCATCCTT | Tm 60ºC | 72 | Reference gene for qPCR | 91; 0.959 |
| β-Tubulin | Tub-F<br>Tub-R | CTGCGGTCGCTATGTTCCT<br>CCCTTGGCCCAGTTGTTTC | Tm 60ºC | 147 | Reference gene for qPCR | 90; 0.995 |

The presence of *Cast_Gnk2-like* gene was verified by PCR in both transcriptional senses employing the specific primers Cast_Gnk2-like-F and Cast_Gnk2-like-R. [1]This fragment includes T-35S region (See Online resource 1). [2] This fragment includes CaMV35S region (See Online resource 1). F: forward; R: reverse.

Transformed lines

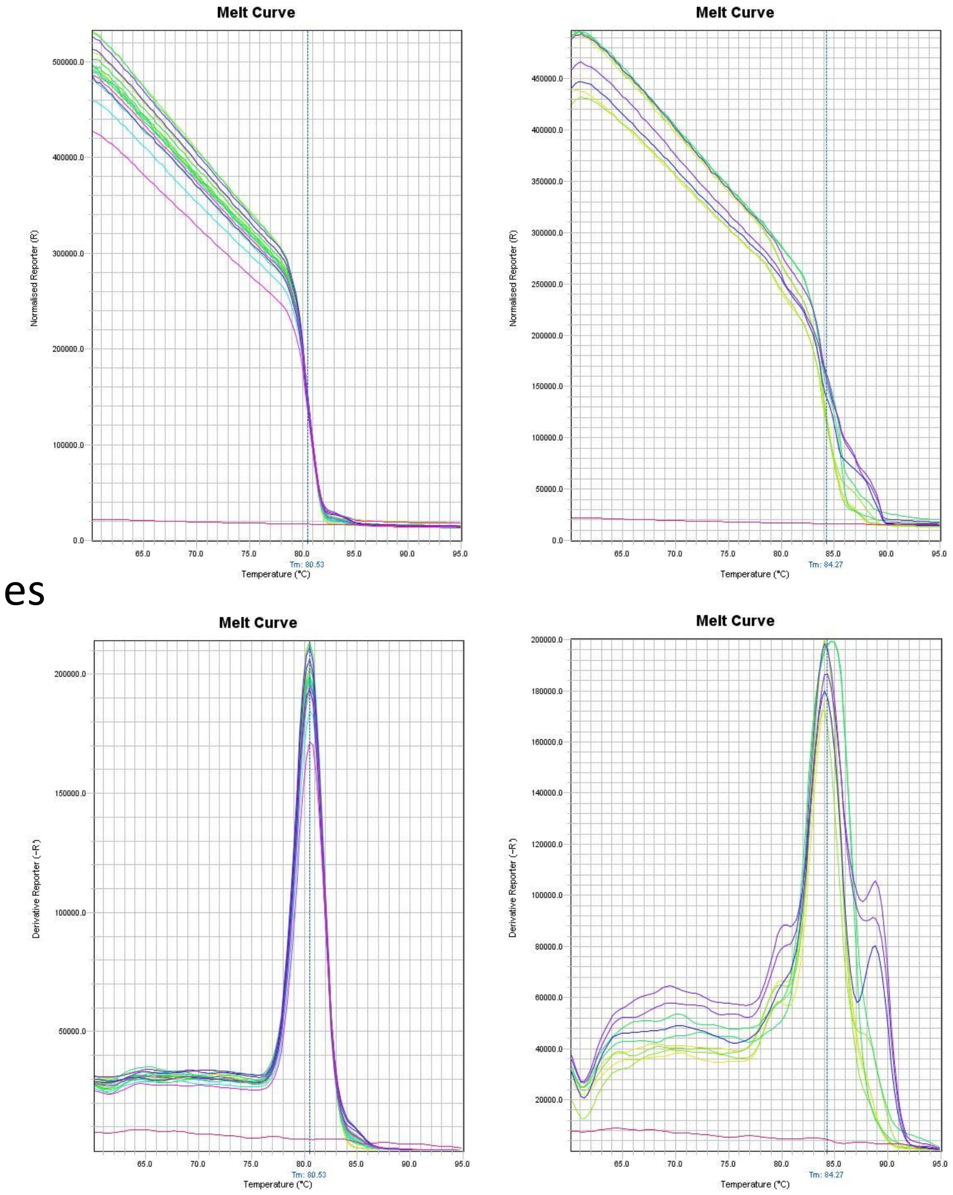


WT

**Online resource 3.** Melt curve analysis of *Q. ilex* transformed lines (left) and WT genotypes (right) upon qPCR reactions with CAMV35S promoter primers. Normalised reporter (up) shows the SYBR green fluorescence variation with the increment of temperature starting from the Tm of the primers, and the abrupt decrease in fluorescence correspondent to the Tm of the amplicon (indicated in the x-axis in blue). Derivative reporter (down) highlights the Tm of the amplicon in the form of a peak. *Q. ilex* transformed lines show a unique peak that corresponds to a unique amplicon. WT genotypes show a Tm of the amplicon distinct from the transformed lines. The low fluorescence pink in all images corresponds to the control without DNA. Images from the melt curve report on the StepOne Software v2.3.

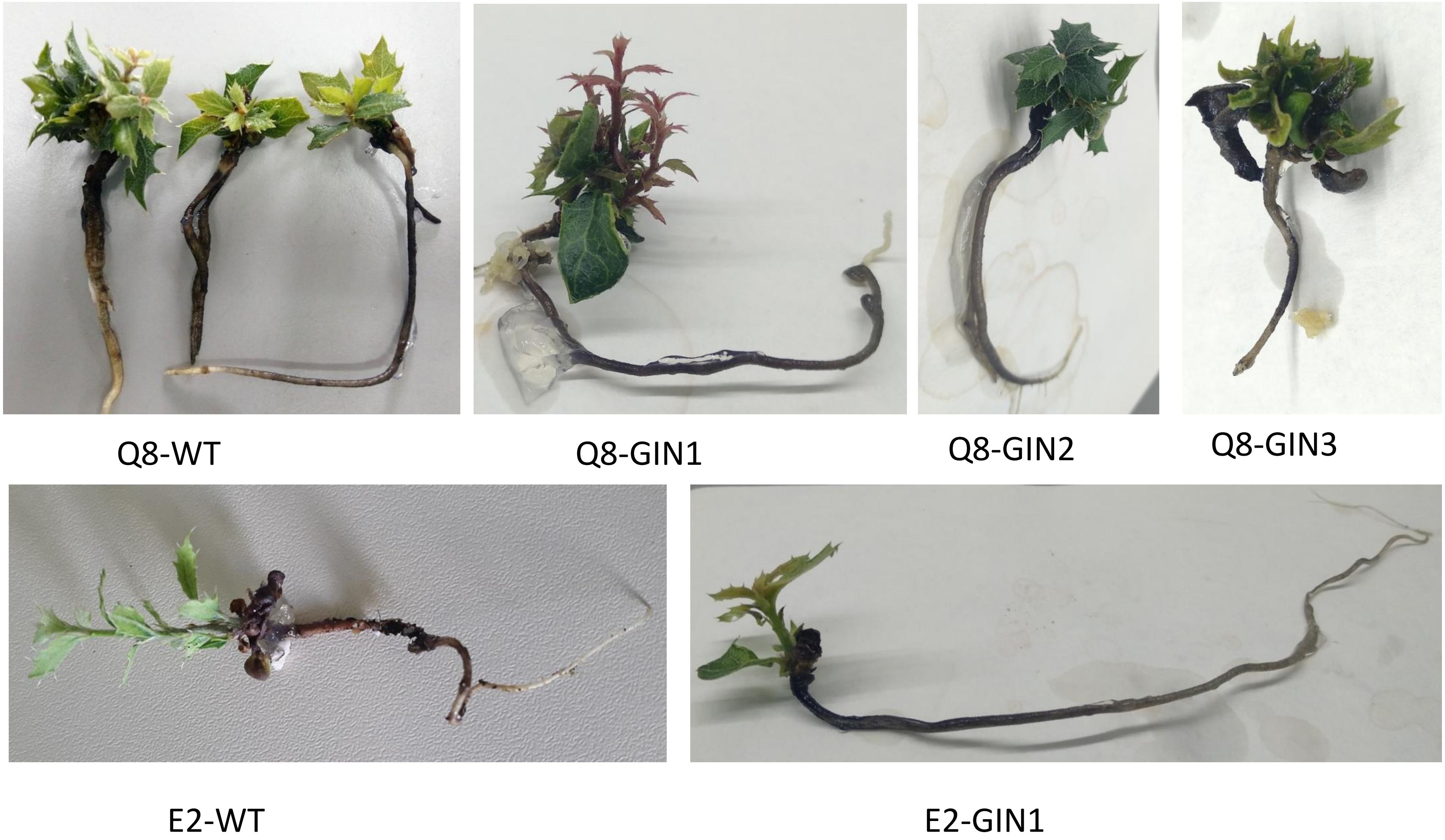


**Online resource 4**. Morphological appearance of non-transformed plantlets and transformed plantlets obtained after embryo germination.
